# TeloNet is born: why all specialities need to be aware of telomere biology disorders

**DOI:** 10.3389/fmed.2026.1780232

**Published:** 2026-04-24

**Authors:** Hilary J. Longhurst, Jane K. Paxton, Hemanth Tummala, Duncan M. Baird, Josu de la Fuente, Austin G. Kulasekararaj, Guruprasad P. Aithal, Jennifer A. Dickens, Pilar Rivera-Ortega, Ramsay Bowden, Joanna Large, Inderjeet Dokal, the TeloNet Group

**Affiliations:** 1 Dyskeratosis Congenita Action (DC Action), London, United Kingdom; 2 Centre for Genomics and Child Health, Blizard Institute, Faculty of Medicine and Dentistry, Queen Mary University of London, London, United Kingdom; 3 Division of Cancer and Genetics, Cardiff University, Cardiff, United Kingdom; 4 Department of Paediatric Haematology, Imperial College London, London, United Kingdom; 5 Department of Immunology and Inflammation Centre for Haematology, Imperial College, London, United Kingdom; 6 Department of Haematology, King's College Hospital, Denmark Hill, London, United Kingdom; 7 NIHR Nottingham Biomedical Research Centre, Nottingham University Hospitals NHS Trust and the University of Nottingham, Nottingham, United Kingdom; 8 Nottingham Digestive Diseases Centre, Translational Medical Sciences, School of Medicine, University of Nottingham, Nottingham, United Kingdom; 9 Department of Medicine, Cambridge Institute for Medical Research, University of Cambridge, Cambridge, United Kingdom; 10 Royal Papworth Hospital, Cambridge, United Kingdom; 11 Department of Respiratory Medicine, Royal Devon and Exeter Hospital, Exeter, United Kingdom; 12 Department of Genomic Medicine, University of Cambridge, Cambridge, United Kingdom; 13 Department of Haematology, Kings College Hospital, London, United Kingdom; 14 Centre for Genomics and Child Health Blizard Institute, Queen Mary University of London, London, United Kingdom

**Keywords:** bone marrow failure, cirrhosis and portal hypertension, dyskeratosis congenita, interstitial lung disease, multidisciplinary care, pulmonary fibrosis (MeSH), telomere - genetics, telomere biology disorder

## Abstract

Telomere biology disorders (TBDs) and short telomere syndromes are difficult to diagnose, requiring a combination of clinical acumen, gene variant analysis and ideally, telomere length. Severe phenotypes include the ultra-rare dyskeratosis congenita and related early-onset syndromes. More commonly, TBDs can present in adulthood with single- or multi-system fibrotic disease, apoptotic bone marrow failure or malignancy. In the general population, shorter telomere lengths are associated with chronic inflammation, fibrotic disorders, cardiovascular disease, malignancy and disorders of ageing. Diagnosis and expert multisystem care are important; TBD-related conditions require non-standard treatments, minimising immunosuppression and potentially profibrotic treatments. All too aware of the challenges TBD patients face and the urgent need for coordinated care, the patients group DC Action brought together patients, medical professionals and scientists: the “TeloNet” alliance, to share best practice and develop diagnostic and management pathways. This mini review describes the first TeloNet meeting, summarising current United Kingdom (UK) practice, in the context of global provision, drawing attention to challenges and improvements required for timely diagnosis, coordinated monitoring and care for people living with TBDs. TeloNet is UK-focussed but the challenges described have relevance across disparate nations and healthcare systems. Those with an interest in TBDs are invited to join TeloNet by contacting info@dcaction.org.

## Introduction and context

Telomere biology disorders are a group of multi-system diseases exhibiting marked clinical and genetic heterogeneity (1–3). Recognition of these disorders came through studies on dyskeratosis congenita (DC) which is now considered to be the paradigm telomere biology disorder. DC is characterized by muco-cutaneous abnormalities (abnormal skin pigmentation, nail dystrophy, leukoplakia), bone marrow failure and a predisposition to malignancy. Bone marrow failure is the principal cause of mortality and patients display features of premature aging.

**Figure 1:**
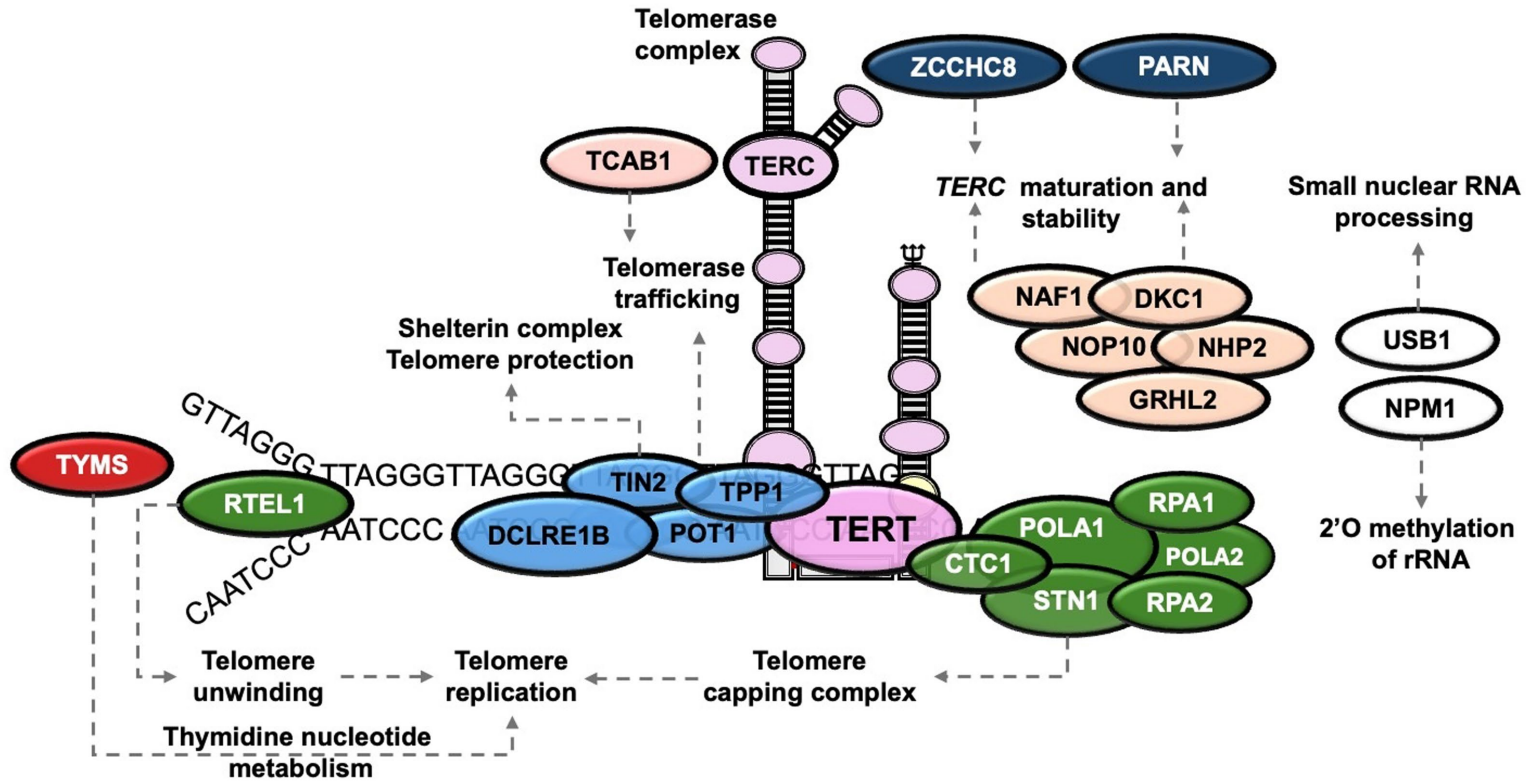
Telomere structure. Proteins subject to gene variants affecting telomerase (TERC, TERT, DKC1, NOP10, NHP2, NAF1), telomerase trafficking (TCAB1), the shelterin complex (ACD/TPP1, POT1, TINF2), or telomere replication (RTEL1, CTC1, POLA1, STN1) or telomere gene processing (ZCCHC8, PARN) are shown.

Studies over the last three decades have led to significant advances with many disease genes [*DKC1* (1998), *TERC* (2001), *TERT* (2005), *NOP10* (2007), *NHP2* (2008), *TINF2* (2008)*, USB1* (2010), *TCAB1* (2011)*, CTC1* (2012), *RTEL1* (2013)*, ACD* (2014)*, PARN* (2015)*, POT1* (2016), *STN1* (2016), *NAF1* (2016), *ZCCHC8* (2019), *NPM1* (2019), *MDM4* (2020), *DCLRE1B* (2022), *RPA1* (2022), *TYMS-ENOSF1* (2022), *POLA1* (2024), *RPA2* (2024), *POLA2* (2024)] having now been characterised (4, 5). Many of these have an important role in telomere maintenance because they encode components that are part of the telomerase holoenzyme (TERC, TERT, DKC1, NOP10, NHP2, NAF1), are important in telomerase trafficking (TCAB1), constitute the shelterin complex (ACD/TPP1, POT1, TINF2), necessary for telomere replication (RTEL1, CTC1, POLA1, STN1) or are required for processing of the telomere genes (ZCCHC8, PARN) (6). These disorders are therefore principally diseases of defective telomere maintenance and patients usually (but not always) have very short and/or abnormal telomeres (Figure 1).

**Figure 2:**
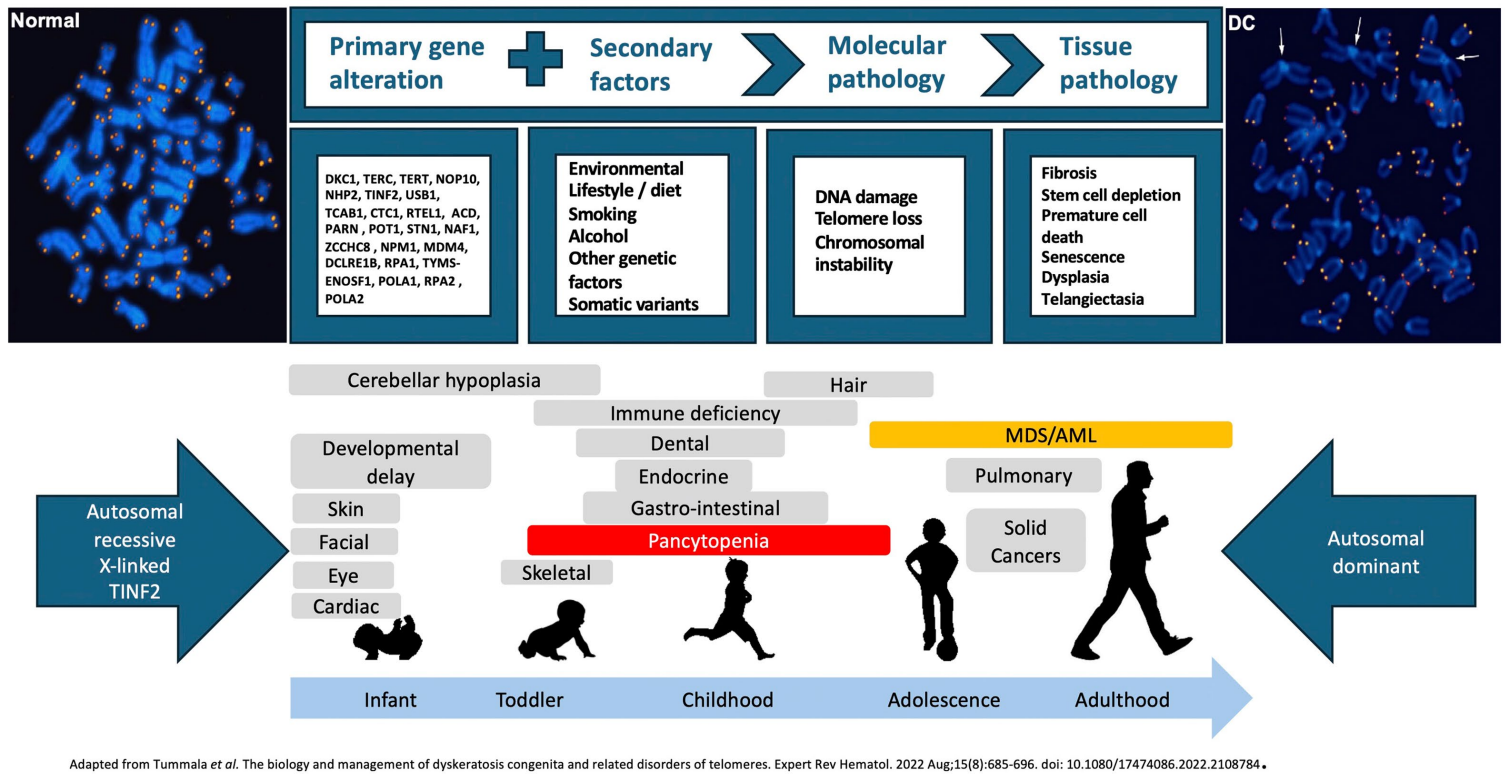
Causes and consequences of telomere biology disorders. Genetic variants associated with impaired telomere maintenance combine with environmental factors to accelerate telomere attrition and cellular consequences of critically short telomeres. Early onset, syndromic or multisystem disease is associated with autosomal recessive, X-linked recessive, or heterozygous TINF2 variants, whereas autosomal dominant inheritance is more commonly associated with later onset, single organ disease. MDS = myelodysplastic syndromes, AML = acute myeloid leukaemia.

The genetic advances have led to the association of dyskeratosis congenita with a number of other diseases. This includes the severe multi-system disorders Hoyeraal-Hreidarsson (1999), Revesz (2008) and Coats plus (2012) syndromes as well as a subset of patients with aplastic anaemia (2002), myelodysplasia (2003), leukaemia (2009), liver disease (2009) and pulmonary fibrosis (2007). This wide spectrum of diseases ranging from syndromic dyskeratosis congenita to aplastic anaemia can now be regarded principally as disorders of defective telomere maintenance - “the telomeropathies,” “short telomere syndromes” or “telomere biology disorders” (Figure 2).

In the clinic, genetic recognition has translated into improvements in diagnosis and clinical outcomes (7). Patients with bone marrow failure arising primarily from defective telomere maintenance show good responses to drugs such as the attenuated androgen danazol rather than conventional immunosuppressive agents employed in idiopathic forms of aplastic anaemia (6). Genetic tests based on next generation sequencing, including Whole Genome/Exome Sequencing (WGS/WES) are proving very helpful in diagnosing patients particularly those with atypical presentations (4).

Study of dyskeratosis congenita and related diseases has shown the importance of telomere maintenance in human physiology including normal haematopoietic function and aging. Defective telomere maintenance arising from germline disease gene variants results in a wide range of clinical phenotypes including syndromic dyskeratosis congenita, bone marrow failure, leukaemia and pulmonary fibrosis – “telomere biology disorders”. These variable clinical presentations mean patients can present to a wide range of clinical specialties. The precise incidence of these disorders is not known; while syndromic dyskeratosis congenita is rare (~1 per million), presentations with bone marrow failure or pulmonary disease are much more common (5, 8–10).

Awareness of TBDs amongst the general public and healthcare providers is low. Patients require lifelong multi-disciplinary monitoring, care and support. The route to diagnosis is often long and difficult and it is thought that significant under-diagnosis is likely (1). Some patients are misdiagnosed and treated with medications that are deleterious. Others have been refused access to care due to lack of clinical knowledge and expertise. At the meeting, three patients gave an emotional account of their long and convoluted routes to diagnosis and the current situation regarding their care.

TeloNet is an informal network of patients, clinicians and scientists with an interest in TBDs convened by DC Action; a UK-based charity. The vision is that TBDs will be recognised by the UK National Health Service (NHS), as multisystem disorders and that the necessary, multidisciplinary specialist care will be available in a number of geographically organised centres to which patients can be referred. At present specialist referral is not straightforward, and existing centres cannot offer MDT care to all who need it.

DC Action advocates for improved services with the NHS. The TeloNet network provides a platform for development of a consensus diagnostic and management pathway with input from all relevant clinical disciplines and the TBD community. As an adjunct, TeloNet aims to facilitate communication, education and sharing of expertise between healthcare professionals in primary, secondary and tertiary care settings resulting in improved services for patients and a platform for clinical studies and research.

TeloNet will be an important vehicle towards the goal of improved diagnosis and harmonized care networks for patients with Telomere Biology Disorders. It will also help facilitate future research with the hope of more targeted and efficacious new therapies.

## Improving diagnosis

Recognition of clinical scenarios where telomere biology disorder (TBD) testing is important is the first step. Children and younger people may have the classical dyskeratosis congenita triad of patchy skin (de)pigmentation, nail dystrophy and oral leukoplakia, but older patients are more likely to present with single organ manifestations, including bone marrow failure/dysplasia, pulmonary fibrosis, steatohepatitis, cirrhosis/ nodular regenerative hyperplasia and early onset oral or other HPV-related malignancy. Cohort studies of individuals with TBDs found an increased cancer risk compared with the general population (9) (Supplementary Figure 1).

Diagnosis can be confirmed by identification of a (likely) pathogenic variant in a telomere-maintenance gene, with or without telomere length measurement (1, 11, 12).

## Genetic diagnosis

The implementation of R91 (haematology), R421 (respiratory medicine) and R15 (immunology) gene panels within NHS England has been instrumental in diagnosing telomeropathy-related bone marrow failure and respiratory syndromes by identifying genetic variant(s) in associated genes (4, 13) (Supplementary Table 1).

However, given the multisystemic nature of TBDs and the possibility of presentation via various specialities, a more complete TBD gene panel is imperative, to provide a comprehensive and multidisciplinary approach to the diagnosis and management of TBDs. A dedicated TBD gene panel would enable genetic evaluation for patients with a personal or family history indicative of TBDs, facilitating timely and accurate diagnoses (14). The ability to identify disease-causing variants in genes associated with telomere dysfunction, will enable clinicians to tailor management strategies and implement surveillance programs specific to the affected gene or variant, reducing morbidity and mortality. Unlike condition-specific panels, a comprehensive TBD gene panel would address the multisystemic nature of these disorders, enhancing coordination of care between specialties. Integration of TBD genes, currently represented in different Genomics England panels, along with flexible and frequent updating as new genes implicated in TBDs are discovered, would ensure that patients receive the benefits of precision medicine while streamlining genetic testing processes. Establishing a TBD gene panel thus represents a critical step towards optimising care pathways, improving outcomes, and advancing the understanding of TBDs across disciplines.

Increasing moves towards whole genome sequencing as the primary methodology for genomic investigations will enable easy integration of TBD gene variant analysis into new and existing panels. Polygenic common variants in telomere genes may also confer risk of TBD of magnitude similar to that of a single pathogenic TBD gene variant. A risk score diagnostic strategy may provide future diagnostic clarity for those in whom no single pathogenic variant is identified (15).

## Analysis of telomere length is critical

Telomere biology disorders exhibit a wide range of clinical manifestations but, in general, they share the common molecular defect of short telomeres, consequently telomere length testing has emerged as a valuable diagnostic tool (16). There are a multitude of methods for estimating telomere length, however reliability and error has limited their clinical application, with two methods, Flow-FISH and high-throughput STELA, which are widely used for diagnosis.

Flow-FISH measures the mean telomere length across all chromosomes, whereas high-throughput STELA measures telomere length of chromosome 17p, which is typically has the shortest telomere. Results are reported as centiles, based on age-related healthy population telomere lengths. Telomere length less than first centile has very high sensitivity and specificity for underlying TBD. Most people with TBD have telomere length in the lowest tenth centile. However, although still a useful diagnostic tool, utility of telomere length testing reduces in older populations and normal telomere length does not completely exclude TBD (17, 18).

Flow-FISH was the first reliable accredited methodology used for clinical diagnosis of telomere biology disorders. It combines flow cytometry with fluorescent in-situ hybridisation (FISH) to measure telomere length in specific leukocyte cell populations. This method uses fluorescently labelled probes that hybridise to telomeric DNA to provide telomere length estimates at the single-cell level. Flow-FISH is accurate and provides powerful clinical information, but because it relies on flow cytometry, is limited in scale and throughput (16–18).

Single Telomere Length Analysis (STELA) has also been adopted as a clinical diagnostic. This accredited technique combines high resolution and low error, with a high-throughput DNA based analysis. STELA is able to measure shorter telomere lengths than other methods. Samples (whole blood or DNA) can be sent via routine laboratory transport (16–18).

STELA thus has the potential to provide accurate clinical telomere length information for diagnostic purposes, from a wide range of tissue types, at the scale needed to address the increasing demand for clinical telomere length testing. STELA and Flow-FISH are ISO accredited; STELA ISO17025, Flow-FISH ISO9001.

Although they are ISO accredited, these important diagnostic tools are not yet commissioned by the Department of Health and Social Care in England and are confined to research laboratories.

## What happens in paediatric care?

Paediatric TBDs most likely represent the severe end of the TBD spectrum (Figure 2). As well as a greater likelihood of presenting with typical dyskeratosis congenita or syndromic features, bone marrow failure is often the first major complication (5, 19). Onset is at median age 10–15 years, compared with later onset of liver complications (30–40 years) or pulmonary fibrosis/emphysema (50–60 years) (19). Thrombocytopenia is the most common manifestation, often resulting in misdiagnosis as idiopathic thrombocytopenia, progressive despite conventional therapy. National Bone Marrow Transplant multidisciplinary meetings have been helpful in minimising diagnostic delay, although the significance of relatively short (>1–10th centile) telomeres without genetic variant or progressive cytopenias remains uncertain.

Reduced intensity conditioning has reduced endothelial and other complications of transplant (19–23), reducing short term (1 year) mortality from around 85% to less than 30% (24).

UK transplant protocols are based on Dietz 2011 (25) namely:

1. Alemtuzumab (day −10 to −6).
2. Cyclophosphamide 50 mg/kg (day −6).
3. Fludarabine 40 mg/m^2^ (day −6 to −2).
4. Total body irradiation 200 cGy (day −1).

With cyclosporin A and mycophenolate graft vs. host prophylaxis.

Access to T cell (TCRab) and B cell (CD19+) depletion effectively extends availability of transplantation to all children: attenuated androgens should not be required pre-transplant but should be considered post-transplant, with the aim of reducing the high medium-term morbidity and mortality (26).

Challenges remain. Molecular clonal screening needs to be mandated, since testing is often declined, owing to unavoidable lack of evidence of benefit in this ultra-rare disorder. Reconstitution is often limited, particularly of T cells (owing to TBD-related thymic dysfunction) and platelets (bone marrow microenvironment dysfunction).

While progress has been made in short term post-transplant survival (70% at 1 year), long-term post-transplant survival of median 6 years urgently needs improvement (24).

In a 150-case series of subjects with TBD, 9 of 42 (21%) with dyspnoea had hepatopulmonary syndrome, often presenting in childhood (median 25 years, range 8–49). This progressed to death or liver transplantation within 6 years (range 4–10). Liver transplantation was curative but did not prevent subsequent pulmonary fibrosis. In contrast, pulmonary fibrosis, with or without emphysema, occurred in 33 cases (79%) and presented in adulthood; median 55 years (range, 40–77) (27).

An additional common, severe paediatric problem is gastrointestinal vascular ectasia. This is particularly common in association with TINF2, TERC, TERT, CTC1 and unknown telomere gene variants and may occur with or without history of transplantation (28).

## What happens in adult haematology?

Telomere Biology Disorders (TBDs) presenting in adulthood or diagnosed in later years are largely under-recognised due to lack of classical features, but their correct identification is of utmost importance. Although improvements in genetic diagnosis of TBDs have been facilitated in UK by establishment of NHS Genomes England test directories (R91, R421, R15), these are not comprehensive. Moreover, availability of screening tests to measure telomere length is lagging. Adult-onset TBDs commonly show telomeres in the 1st to 10th percentile for age. However, normal telomere length does not exclude TBD and some cases may not have an identifiable genetic cause. TBD genetic aetiology includes all modes of inheritance, with autosomal dominant the most frequent in adult-onset disease. Variable symptom onset due to incomplete penetrance, variable expressivity, and genetic anticipation add to the diagnostic challenges.

In the UK, unfortunately there is no defined pathway for investigation and management of patients with genetic bone marrow failure, although aplastic anaemia guidelines have recently been published (29). There is a lack of knowledge and expertise, in view of the rarity of diagnosed TBDs in adults. Affected patients are faced with numerous complications, including increased risk of lung fibrosis, liver disease and cancers, requiring close surveillance, multidisciplinary monitoring and management (1). Varied presentation with ‘no’ haematological phenotype is also a major limitation in investigation of TBD patients. The lack of transition clinics and regular/planned monitoring strategy restricts the ability to properly monitor even diagnosed TBD patients into late adolescence and adulthood, looking at other organ dysfunction (liver, lung, endocrine, cardiac, fertility, bone health, cancer) or providing psychological support. This is further compounded by lack of established funding or commissioning; required to streamline pathways.

Monitoring of bone marrow for somatic mutations, including Chr1q + and U2AF1S34 can identify those at risk of haematological progression (30). Severe aplastic anaemia, myelodysplasia or myeloid malignancy are common occurrences. Somatic rescue can occur but is uncommon (30, 31). Non-myeloablative conditioning regimens, with better control of short telomere-related, infectious and thrombotic complications means that transplantation is increasingly undertaken for adults. Nevertheless, for many, transplantation is not feasible.

Danazol and other androgens, particularly oxymetholone, have proven effective in observational trials for treatment, improving both telomere lengths and blood counts, in many cases allowing medium to long-term stability (26, 32, 33). Androgens may also be effective for non-haematological complications- but prospective trials have been disappointing.

Outstanding important questions include:

1. Are low dose androgens effective? (34)
2. Are androgens with a better tolerability profile, such as tibolone, stanozolol or oxandrolone effective?
3. Is earlier, or even prophylactic treatment with androgens effective and would this allow low dose treatment which could be well tolerated for the longer term? (34)

## Lung involvement

Approximately one third of monogenic familial pulmonary fibrosis (PF) is due to a telomere biology disorder (TBD) (35, 36). Identifying individuals with a telomeropathy is crucial for their optimal care, both in terms of identifying extra-thoracic disease and in tailoring their PF management. Emerging evidence on the potential increased risk of standard immunosuppression regimes (both in PF management and post-transplant) mean personalised treatment is increasingly important (37–39). Conversely, antifibrotics are likely to be beneficial and should not be withheld due to the ‘non-idiopathic’ nature of telomere biology disorder-related PF (40, 41).

The introduction of a standardised genetic testing panel for familial PF (R421) in the genomic test directory allows identification of pathogenic variants in many genes involved in telomere maintenance including TERT, the commonest PF-associated gene. Though access to this test is universal, it remains underutilised outside of the three dedicated familial PF centres in Cambridge, Exeter and Manchester and some other major interstitial lung disease centres. This in part reflects a lack of awareness of, and confidence in, requesting and interpreting results and highlights an unmet need within respiratory medicine for standardising care in this area.

The development of collaborative networks between respiratory and other relevant specialities is needed to optimise care nationwide as well as to facilitate much-needed research in familial PF due to TBDs.

## What happens in hepatology?

Liver receives a dual blood supply from the hepatic artery as well as the portal vein. Portal blood exposes the liver to environmental stressors such as alcohol, food and bacterial toxins. Clinically evident liver injury is a manifestation of TBDs. In genetically confirmed telomeropathies, 10–40% of patients have evidence of liver involvement (42, 43).

Liver manifestations range from raised liver enzymes, drug/alcohol-induced liver injury, hepatosteatosis, nodular regenerative hyperplasia, cirrhosis to hepatopulmonary syndrome. Management of liver manifestations depends upon the degree of liver fibrosis and portal hypertension, as well as involvement of other organs, most commonly the respiratory system (44–46).

Diagnosis of telomeropathy in people with liver manifestations depends upon clinical suspicion. There is no panel of genetic tests or pathway specified in the UK genomic directory when telomere biology disorder is suspected in a person presenting with liver manifestations. Harmonising genetic test panels to identify telomere biology disorders in individuals presenting with a range of manifestations involving different organ systems is a priority.

## Multi-disciplinary clinics: the Cambridge multi-disciplinary team (MDT)

The multisystem nature of telomere biology disorders means that multi-disciplinary team (MDT) management is critical to optimise patient care. Diagnosis is often the first challenge. The broad range of presentations and a lack of recognition means these disorders are almost certainly underdiagnosed. The range of medical and surgical specialities to which patients can present includes respiratory medicine, haematology, dermatology, hepatology, immunology, maxillofacial surgery, paediatrics, clinical genetics and others.

Discussion between specialists in an MDT format is crucial for initiating appropriate diagnostic investigations to correctly identify patients with a telomere biology disorder. For patients with an established diagnosis, the MDT is important for optimising management. Liaison between specialists is key to making sure that complications of the disorder, not apparent on initial diagnosis, are identified and treated. Clear pathways for referral between specialties and standardised guidelines on appropriate management of symptomatic and pre-symptomatic individuals should be developed. Clinicians from relevant specialities can share best practice in their field with the rest of the team. MDT care benefits patients in ensuring the disorder is managed holistically. In-person or virtual MDT clinics, where patients are seen by multiple specialists in one clinical setting reduce hospital visits, enabling joined-up care. MDTs also facilitate patient data being collected in a standardised format to open up research opportunities (47).

In Cambridge, work across two hospital trusts (Royal Papworth Hospital NHSFT and Cambridge University Hospitals NHSFT), brings together representatives from all major specialities involved in the care of telomere biology disorder patients to form an MDT. The group currently meets in person on a quarterly basis to discuss patient cases and share best working practices. This has resulted in changes to patient management and greater referrals between specialities. The number of relatives referred for cascade screening has also increased. Based on the success of the current model the group plan to increase the frequency of MDT meetings and to establish an in-person MDT clinic to further improve coordinated care. The TeloNet meeting provided an ideal opportunity for clinicians from the Cambridge MDT to forge links with clinicians and scientists from across the UK.

Globally, provision for TBD patients is expanding, albeit still limited. In 2024 Team Telomere, a US-based patient organisation, launched a Centers of Excellence programme to improve access to and ensure standardisation of specialised care, with accredited centres in the USA, Europe and Israel.^1^

## The role of the clinical nurse specialist in telomere biology disorders

Being diagnosed with a rare disease poses challenges; emotional and logistic as well as physical. Patients can feel bewildered, alone and unsure, needing support and continuity. The role of the nurse specialist is crucial in all these respects and nurses are often key coordinators of complex care needs (48).

The UK rare disease framework outlines priorities of increased awareness, faster diagnosis, better coordination of care and improved access to specialist care, including treatment and drugs (49).

Access to nurse specialists with a deep understanding of the patient pathway is essential from the first visit through diagnosis, family planning, admission for treatment and beyond. Nurses are a key point of contact, information providers and a source of support and reassurance. They are skilled in making the complex understandable, and in arming patients with the language and essential confidence to take agency in their own care.

Because they understand patient needs, nurses are well placed to design and shape services.

They are key co-ordinators working with multiple stakeholders including the wider medical teams, other service providers, patient support and advocacy groups. They offer a value-for-money holistic service working clinically and strategically to support patients and their families.

In the context of TBD care, specialist nurses are often clinic- and therefore speciality-based (50).

They provide additional patient support and co-ordination of services, including facilitation or research and multidisciplinary team discussions. However, there is great need for pan-speciality expansion of their role.^2^

## Discussion: practical implications for the future

### Research: building an evidence base for prevention and treatment

In order to widen therapeutic options, research needs to be embedded into specialist clinics ensuring universal access to treatment. Repurposed drugs and access to newer genetic therapies need to be available, with reporting of outcomes via collection of routinely available NHS data. Platform-style, multifactorial studies with Bayesian randomisation have proved pragmatic and useful in other settings (51–54).

The development of Biobanks is essential to allow investigation of mechanisms underlying telomere attrition and to mitigate the current difficulties experienced by scientists in access to appropriate biological material. DC Action/ TeloNet favours an inclusive “opt out” approach, with opportunities for multisource (General practitioners (GPs), local hospitals, patients) input, co-ordinated by the specialist centre.

### Registers and databases

There is a dearth of natural history of disease data which needs to be addressed perhaps by country specific registers that cooperate to ensure similar data is collected.

The US organsiation, Team Telomere, is partnered with RareX, a patient led register. Patients from any country are free to add their data. The objective is to make patient information available to researchers.^3^

The Clinical Care Consortium of Telomere-Associated Ailments (CCCTAA) is compiling a repository of coded clinical data on patients with Telomere Biology Disorders (TBDs) submitted by researchers from CCCTAA member institutions. The study is managed by the US National Cancer Institute with the objective of facilitating collaborative research across CCCTAA member institutions.^4^

### Clinical guidelines

Team Telomere has published two iterations of Telomere Biology Disorders Diagnosis and Management Guidelines, the latest in 2022. This consensus-level guidance, with contributions from experts in Telomere Biology Disorders, is widely referred to by both patients and healthcare professionals. It is, however, not an officially endorsed guideline.^5^

To meet the needs of TBD patients in the UK, alongside MDT provision in specialist centres, nationally pragmatic guidelines, incorporating community services as well as multi-disciplinary specialist clinics, are needed.

### Call to action

Telomere biology disorders (TBDs) present at any age. They require a high degree of awareness from a variety of clinicians, including haematologists (bone marrow failure), pulmonologists (interstitial lung disease, hepatopulmonary syndrome), hepatologists (steatohepatitis, portal hypertension, cirrhosis), gastroenterologists (vascular ectasia, strictures, inflammatory bowel disease) and oral medicine specialists (oral leukoplakia, oral/oesophageal cancers), ophthalmologists (retinal exudates, lacrimal gland strictures), paediatricians (developmental delay and syndromic features), immunologists (T cell dysfunction, HPV-related disease, combined and common variable immunodeficiencies), geneticists, general medics and general practitioners.

- Diagnostic pathways (genetic panels, ideally on a WGS platform to allow future re-curation and telomere length testing) need to be more widely available, along with a multigenic risk assessment, when available and validated.
- Care for those affected and their at-risk relatives needs to be delivered by multidisciplinary specialist clinics, using remote consultation where necessary to ensure equitable access.
- Data collection and sharing, and tissue banking needs to be built into specialist services in a way that allows equitable local access to research and development of therapeutic options.

Co-ordinated diagnosis and care will ensure that people living with TBDs delay or prevent onset of complications, remaining healthier and productive for longer.

Knowledge gained from care of those with telomere disorders has wider implications. Telomere attrition occurs during normal ageing and may occur prematurely in those suffering long term inflammatory disorders (5, 55). Demographic changes in high income countries have necessitated extension of the health-span. Mitigation of age- or chronic disease-related telomere attrition could be a powerful public health strategy in this respect (56).

TeloNet will be an important vehicle towards the goal of improved diagnosis and harmonized care networks for patients with TBDs. Future cohorts of clinicians will be encouraged to develop an interest in TBDs.

It will also help facilitate future research with the hope of more targeted and efficacious new therapies.

We invite all interested clinicians, allied healthcare professionals, scientists and patients to join the TeloNet community by contacting info@dcaction.org.

## Footnotes

1 https://teamtelomere.org/centers-of-excellence/ (accessed February 2026).

2 https://geneticalliance.org.uk/wp-content/uploads/2024/01/Coordinating-Care-Report-2023.pdf and https://geneticalliance.org.uk/wp-content/uploads/2025/02/RDUK-Care-Coordinator-Report.pdf (accessed February 2026)

3 https://teamtelomere.org/resources/rare-x/ (accessed February 2026). https://rare-x.org/tbd-dc/ (accessed February 2026).

4 https://www.centerwatch.com/clinical-trials/listings/NCT05436431/clinical-care-consortium-of-telomere-associated-ailments-ccctaa-database (accessed February 2026).

5 https://teamtelomere.org/diagnosis-management-guidelines/ (accessed February 2026).

## Group member of the TeloNet Group

Guruprasad Aithal, Nottingham University Hospitals and University of Nottingham; Mohsin Badat, Queen Mary, University of London and Royal London Hospital, London; Duncan Baird, University of Cardiff and TeloNostiX; Shadi Basyuni, Addenbrooke’s Hospital and University of Cambridge; Mandy Bellamy, DC Action; Ramsay Bowden, Addenbrooke’s Hospital and University of Cambridge; Veryan Codd, University of Leicester; John Conway, Exeter Patients in Collaboration for Pulmonary Fibrosis (EPIC) and DC Action; Charlotte Crook, Royal Devon and Exeter Hospital; Bob Dalgleish, Fanconi Hope; Jeannie Dalgleish, Fanconi Hope; Nirupa Desai, Addenbrooke’s hospital and University of Cambridge; Jennifer Dickens, Addenbrooke’s Hospital and University of Cambridge; Giles Dixon, University of Exeter; Inderjeet Dokal, Queen Mary, University of London; Anna Duckworth, EPIC, DC Action and University of Exeter; Victoria Elworthy, Royal Devon and Exeter Hospital, Exeter; Josu de la Fuente, St Mary’s Hospital and Imperial College, London; Claire Garrity-Yates, DC Action; Craig Garrity-Yates, DC Action; Simon Holden, Addenbrooke’s Hospital, Cambridge; Deena Iskander, St Mary’s Hospital, Imperial College, London; Gisli Jenkins, Imperial College, London; Austin G Kulasekararaj, King’s College Hospital, London; Steve Jones, Action for Pulmonary Fibrosis, UK and European Pulmonary Fibrosis Federation; Jackie King, DC Action; Joanna Large, King’s College Hospital, London; Sarah Lines, Royal Devon and Exeter Hospital; Bill Longhurst, DC Action; Jo Longhurst, DC Action; Stefan Meyer, Royal Manchester Children’s Hospital and University of Manchester; Hanya Mahmood, University of Sheffield; Vivian Jean-Marc Michel, DC Action; Deborah Morris-Rosendahl, Royal Brompton Hospital; Jeremy Shanika Nayagam, King’s College Hospital, London; Clara Novo, Crick Institute, London; Helen Parfrey, Papworth Hospital, Cambridge; Phyllis Paterson, Addenbrooke’s Hospital Cambridge; Jane Paxton, DC Action; Laurence Pearmain, Wythenshawe Hospital and University of Manchester; Maria Piggin, PNH Support, UK and PNH Global Alliance; Anna Reed, Royal Brompton and Harefield Hospital, London; Muhammed Riaz, DC Action; Dr. Pilar Rivera-Ortega, Royal Devon and Exeter Hospital and University of Exeter; Brittney Savoni, King’s College Hospital, London; Chris Scotton, University of Exeter; Stefan Stanel, Royal Devon and Exeter Hospital and University of Exeter; Hemanth Tummala, Queen Mary, University of London; Dries Verhoef, DC Action; Bavithra Vijayakumar, Wythenshawe Hospital, Manchester; Alan J. Warren, Addenbrooke’s Hospital and University of Cambridge; Jemma Williams, DC Action.

## Supplementary material

Supplementary Figure 1 Telomere Biology Disorders can affect many organs.

Supplementary Table 1 Telomere-associated genes included in Genomes England Diagnostic Panels.
