# Supplementary figures and images for: TeloNet is born: why all specialities need to be aware of telomere biology disorders
Source: Front Med (Lausanne). 2026 Apr 24;13:1780232. doi: 10.3389/fmed.2026.1780232 (PMC13154602; doi:10.3389/fmed.2026.1780232)

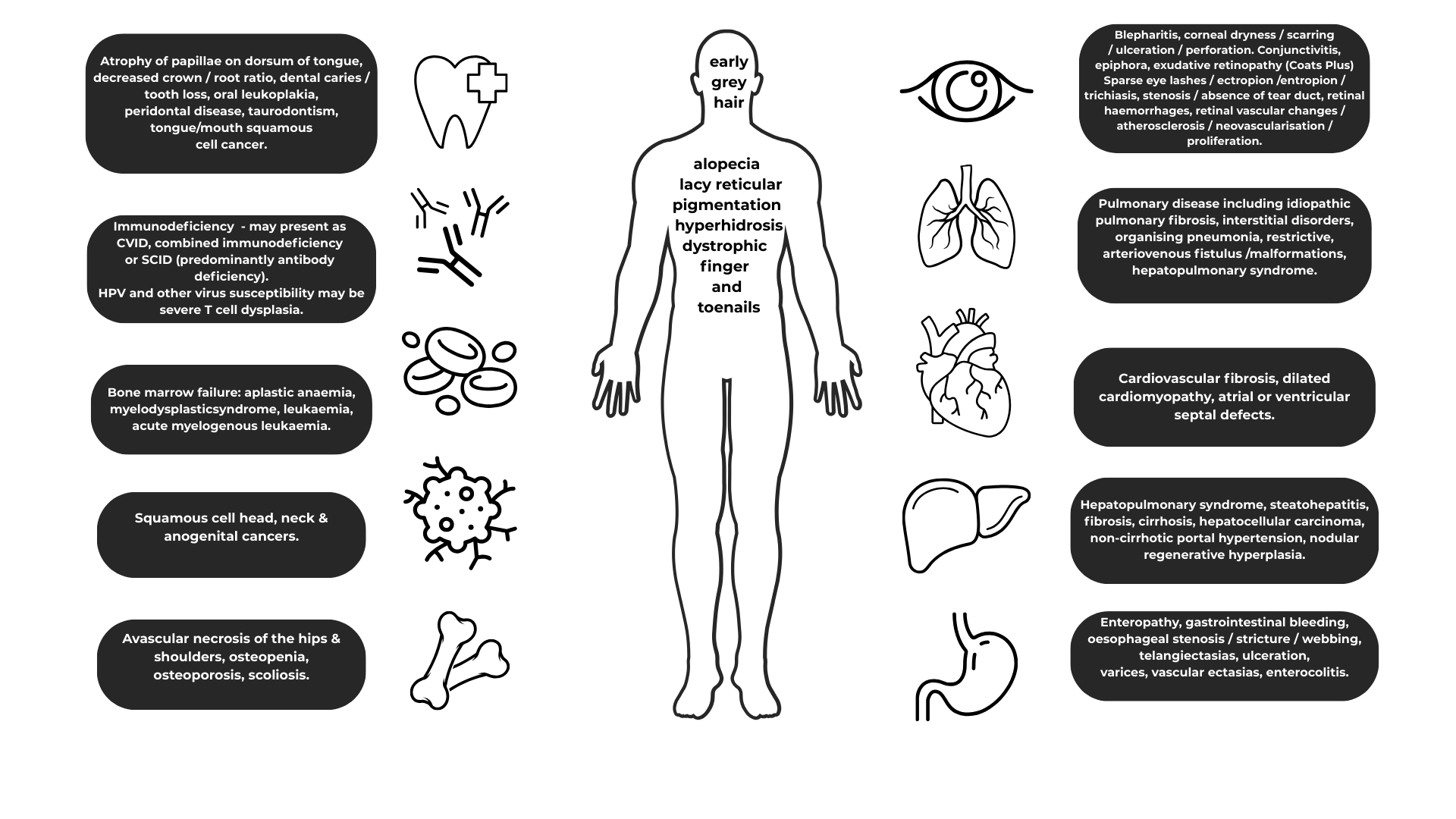

Supplement: Supplementary Figure 1 — Telomere Biology Disorders can affect many organs. [file Image_1.JPEG]
