# Supplementary material for: TeloNet is born: why all specialities need to be aware of telomere biology disorders
Source: Front Med (Lausanne). 2026 Apr 24;13:1780232. doi: 10.3389/fmed.2026.1780232 (PMC13154602; doi:10.3389/fmed.2026.1780232)
Supplement: Supplementary Table 1 — Telomere-associated genes included in Genomes England Diagnostic Panels. [file Data_Sheet_1.PDF]

TBD Associated Gene	Green in R91	Green in R421	Green in R15	Green in other Genomes England panels https://panelapp.genomicsengland.co.uk/
**DKC1**<br>(dyskerin [DKC1])	Yes	Yes	Yes	Haematological malignancies cancer susceptibility. Ataxia and cerebellar anomalies - narrow panel. DDG2P (a component of super panel R27 Paediatric disorders). Pigmentary skin disorders. Fetal anomalies. Intellectual disability.
**NAF1**<br>(nuclear assembly factor 1 ribonucleoprotein [NAF1])	No	Yes	No	Haematological malignancies cancer susceptibility.
**NHP2**<br>(NOLA2 nucleolar protein family A, member 2 [NHP2])	Yes	Yes	Yes	Haematological malignancies cancer susceptibility. Childhood solid tumours cancer susceptibility. DDG2P.
**NOP10**<br>(NOLA nuclear protein family A, member 3 [NOP10])	No	Yes	No	Haematological malignancies cancer susceptibility. DDG2P.
**TERT**<br>(telomerase reverse transcriptase [TERT])	Yes	Yes	No	Childhood solid tumours. Intestinal failure or congenital diarrhoea. Pigmentary skin disorders. Inherited predisposition to acute myeloid leukaemia (AML). Childhood solid tumours cancer susceptibility. Adult solid tumours cancer susceptibility. Haematological malignancies cancer susceptibility. Skeletal dysplasia. DDG2P. Ataxia and cerebellar anomalies - narrow panel.
**TERC**<br>(encodes an RNA: hTR, human telomerase RNA component [TERC])	Yes	Yes	No	Childhood solid tumours. Pigmentary skin disorders. Inherited predisposition to acute myeloid leukaemia (AML). Adult solid tumours cancer susceptibility. Haematological malignancies cancer susceptibility. DDG2P.
**ACD**<br>(telomere protection protein 1 [TPP1])	Yes	Yes	Yes	Haematological malignancies cancer susceptibility.
**TINF2**<br>(TERF1 [TRF1]-interacting nuclear factor 2 [TIN2])	Yes	Yes	No	Ataxia and cerebellar anomalies - narrow panel. Pigmentary skin disorders. Haematological malignancies cancer susceptibility. Cerebellar hypoplasia. Fetal anomalies. DDG2P. Retinal disorders.
**CTC1**<br>(conserved telomere maintenance component 1 [CTC1])	Yes	Yes	No	White matter disorders and cerebral calcification - narrow panel. Haematological malignancies cancer susceptibility. Fetal anomalies. Retinal disorders. DDG2P.
**PARN**<br>(poly(A)-specific ribonuclease [PARN])	Yes	Yes	Yes	Haematological malignancies cancer susceptibility. Fetal anomalies. DDG2P. Intellectual disability.
**RTEL1**<br>(regulator of telomere elongation helicase 1 [RTEL1])	Yes	Yes	Yes	Infantile enterocolitis & monogenic inflammatory bowel disease. Gastrointestinal epithelial barrier disorders. Childhood solid tumours. Haematological malignancies cancer susceptibility. DDG2P.
**WRAP53**<br>(telomere Cajal body associated protein 1 [TCAB1])	Yes	No	No	Childhood solid tumours. Pigmentary skin disorders. Adult solid tumours cancer susceptibility. Haematological malignancies cancer susceptibility. Fetal anomalies. DDG2P.
**RPA1**<br>(replication protein A1 [RPA1])	Yes	No	No	
**POT1**<br>(protection of telomeres 1 [POT1])	No	No	No	Familial melanoma. Li Fraumeni Syndrome.
**STN1**<br>(STN1 subunit of CST- complex subunit [STN1])	Yes	No	No	White matter disorders and cerebral calcification - narrow panel. Haematological malignancies cancer susceptibility. DDG2P. Paediatric disorders - additional genes. Retinal disorders.
**DCLRE1B**<br>(DNA cross-link repair 1B, Apollo)	No	No	No	
**ZCCHC8**<br>(zinc finger CCHC- type containing 8 [ZCCHC8])	No	Yes	No	DDG2P.
**MDM4**<br>(MDM4 regulator of p53 [MDM4])	No	No	No	
**NPM1**<br>(nucleophosmin/ nucleoplasmin family member 1 [NPM1])	No	No	No	DDG2P.
**TYMS-ENOSF1 / TYMS**<br>(thymidylate synthase, antisense; rts [TYMS / ENOSF1])	No	No	No	
**POLA1**<br>(DNA polymerase [POLA1])	No	No	Yes	DDG2P. Intellectual disability
**POLA2**<br>(accessory subunit of DNA polymerase α (polα)/primase [POLA2])	No	No	No	
**RPA2**<br>(replication protein A1 [RPA1])	No	No	No	
**USB1**<br>(U6 snRNA biogenesis phosphodiesterase 1 [USB1])	Yes	No	Yes	Pigmentary skin disorders. DDG2P

A gene with a green listing in a Genomes England panel has a high level of evidence supporting its association with a specific human disorder.

R91 - Cytopenia - NOT Fanconi anaemia https://panelapp.genomicsengland.co.uk/panels/519/

R421 - Pulmonary fibrosis familial https://panelapp.genomicsengland.co.uk/panels/1174/

R15 - Primary immunodeficiency or monogenic inflammatory bowel disease https://panelapp.genomicsengland.co.uk/panels/398/
